# Distinct Roles of Histone Lysine Demethylases and Methyltransferases in Developmental Eye Disease

**DOI:** 10.3390/genes14010216

**Published:** 2023-01-14

**Authors:** Linda M. Reis, Huban Atilla, Peter Kannu, Adele Schneider, Samuel Thompson, Tanya Bardakjian, Elena V. Semina

**Affiliations:** 1 Department of Pediatrics and Children’s Research Institute, Medical College of Wisconsin and Children’s Wisconsin, Milwaukee, WI 53226, USA; 2 Department of Ophthalmology, School of Medicine, Ankara University, 0600 Ankara, Turkey; 3 Department of Medical Genetics, University of Alberta, Edmonton, AB T6G 2R3, Canada; 4 Einstein Medical Center Philadelphia, Philadelphia, PA 19141, USA; 5 Department of Ophthalmology and Visual Sciences, Medical College of Wisconsin, Milwaukee, WI 53226, USA

**Keywords:** histone lysine demethylase, histone lysine methyltransferase, KMT2D, KMT2C, SETD1A, KDM6A, KDM5C, Peters anomaly, Axenfeld-Rieger syndrome, developmental eye disease

## Abstract

Histone lysine methyltransferase and demethylase enzymes play a central role in chromatin organization and gene expression through the dynamic regulation of histone lysine methylation. Consistent with this, genes encoding for histone lysine methyltransferases (KMTs) and demethylases (KDMs) are involved in complex human syndromes, termed congenital regulopathies. In this report, we present several lines of evidence for the involvement of these genes in developmental ocular phenotypes, suggesting that individuals with structural eye defects, especially when accompanied by craniofacial, neurodevelopmental and growth abnormalities, should be examined for possible variants in these genes. We identified nine heterozygous damaging genetic variants in *KMT2D* (5) and four other histone lysine methyltransferases/demethylases (*KMT2C*, *SETD1A/KMT2F*, *KDM6A* and *KDM5C*) in unrelated families affected with developmental eye disease, such as Peters anomaly, sclerocornea, Axenfeld-Rieger spectrum, microphthalmia and coloboma. Two families were clinically diagnosed with Axenfeld-Rieger syndrome and two were diagnosed with Peters plus-like syndrome; others received no specific diagnosis prior to genetic testing. All nine alleles were novel and five of them occurred de novo; five variants resulted in premature truncation, three were missense changes and one was an in-frame deletion/insertion; and seven variants were categorized as pathogenic or likely pathogenic and two were variants of uncertain significance. This study expands the phenotypic spectra associated with KMT and KDM factors and highlights the importance of genetic testing for correct clinical diagnosis.

## 1. Introduction

Members of the histone lysine methyltransferase and demethylase families function in opening and closing chromatin to control gene expression. Heterozygous truncating and loss-of-function missense variants in many members of these families have been associated with human disease, termed congenital regulopathies [1]. Variants in *KMT2D* and *KDM6A* result in Kabuki syndrome with dysmorphic facial features (long palpebral fissures, depressed nasal tip, and large ears), short stature, intellectual disability, hypotonia, skeletal anomalies including hip joint dislocation, abnormal finger pads, genitourinary malformation, immune deficiency, feeding disorders and congenital heart defects [2,3]. Kleefstra syndrome 2 is a rare condition caused by variants in *KMT2C* with moderate to severe cognitive impairment, autism, hypotonia, short stature and mild and variable dysmorphic facial features [4]. Variants in *SETD1A (KMT2F)* cause a neurodevelopmental phenotype with intellectual disability (typically mild), hypotonia, behavioral/psychiatric abnormalities, gastrointestinal anomalies, recurrent infections and dysmorphic craniofacial features including high forehead, ear anomalies and down-slanting palpebral fissures [5]. Claes-Jensen syndrome is an X-linked disorder caused by variants in *KDM5C (JARID1C)* which can affect both males and females with intellectual disability, seizures, short stature and craniofacial features; due to X-inactivation, only half of female carriers are affected [6,7]. 

Common features of these congenital regulopathies include cognitive impairment, craniofacial dysmorphism and short stature, underscoring the importance of histone regulation in the development of brain and craniofacial structures as well as prenatal/postnatal growth. The complex phenotypes associated with histone lysine methyltransferases and demethylases correspond with their broad effects on gene transcription and widespread expression, including in the developing ocular structures. Consistent with this expression pattern, we identified nine cases within our cohort of individuals with developmental ocular anomalies carrying genetic variants in *KMT2D* (five families) and four other histone lysine methyltransferases and demethylases (*KMT2C, SETD1A (KMT2F), KDM6A* and *KDM5C*), thus expanding the phenotypic spectra associated with these genes and suggesting distinct roles in human eye development.

## 2. Materials and Methods

This study presents a subset of patients enrolled into a genetic study of ocular disorders approved by Institutional Review Boards at Children’s Wisconsin, Einstein Medical Center Philadelphia and University of Iowa. Written informed consent including research analysis and photo publication (if applicable) was obtained for every participant. Exome sequencing was undertaken by Psomagen (Rockville, MD) and analyzed using VarSeq (Golden Helix, Bozeman, MT). In silico analysis of variants of interest included filtering for frequency <0.001 in the general population in gnomAD v2.1.1 [8] and for predicted effect upon the protein with CADD phred hg19 and REVEL utilized for missense predictions. Population specific databases were also utilized including Turkish Variome and Iranome [9,10]. Samples were first analyzed for variants in known microphthalmia, anophthalmia, coloboma and anterior segment dysgenesis genes as previously described [11,12]. Analysis of trio or singleton exomes for negative cases identified pathogenic or likely pathogenic heterozygous variants in *KMT2D* (NM_003482.4), *KMT2C* (NM_170606.2), *SETD1A* (*KMT2F*; NM_014712.3), *KDM6A* (NM_021140.4) and *KDM5C* (NM_004187.5) as well as variants of uncertain significance in *KMT2D*. Variant locations were annotated to human Genome Build hg19 and evaluated according to ACMG/AMP guidelines [13].

## 3. Results

### 3.1. Genetic Variants Identified in Histone Lysine Methyltransferases Genes, KMT2D, KMT2C and SETD1A (also Known as KMT2F)

**Figure 1:**
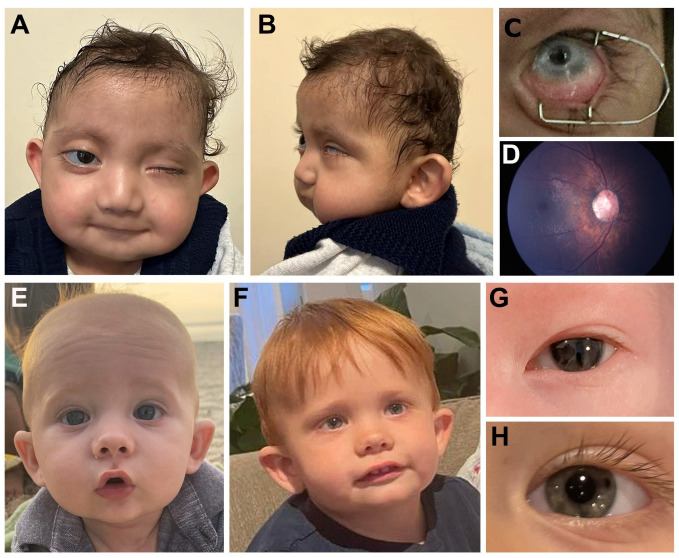
**Clinical images.** (**A**–**D**). Individual 2 with *KMT2D* pathogenic variant showing microcephaly, smooth philtrum, thin upper lip and low-set cupped ears (**A**,**B**), left eye microphthalmia and sclerocornea (peripheral) (**C**), and right fundus photograph showing anomalous optic nerve (**D**). (**E**–**H**). Individual 7 with *SETD1A* pathogenic variant showing high forehead, depressed nasal bridge and cupped ears at 4 months (**E**) and 2 years (**F**) of age along with ocular photos showing slit pupil at 4 days of age (**G**) which had resolved by 2 years of age (**H**).

**Figure 2:**
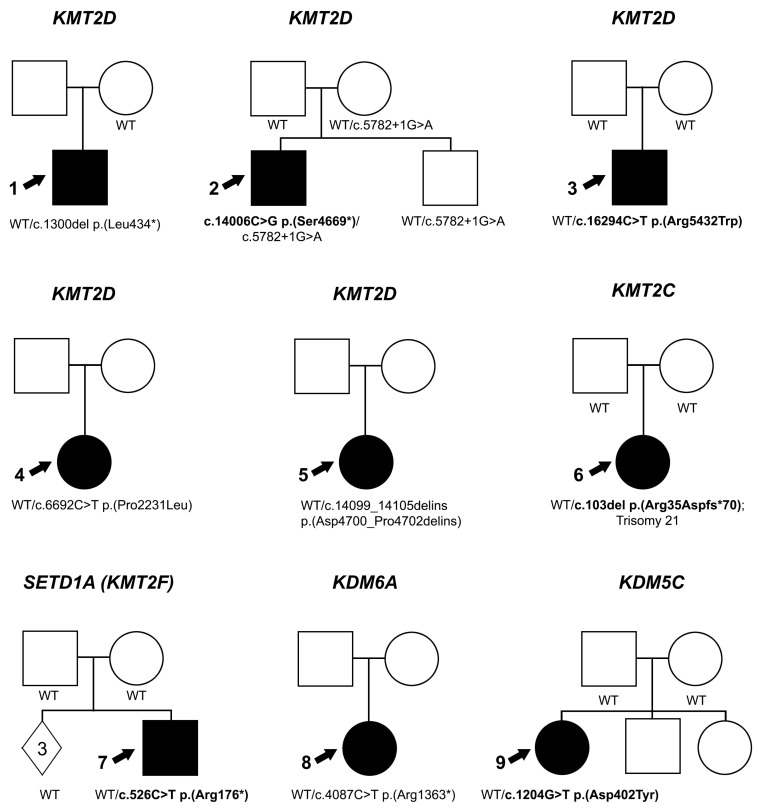
**Pedigree information.** Pedigrees for Individuals 1–9 (indicated with an arrow). Genetic data are indicated for each individual in the family who was tested. De novo variants are indicated in bold; WT indicates wild type sequence at variant location and solid symbol indicates affected individuals.

Seven variants of histone lysine methyltransferases genes were identified in unrelated families affected with anterior segment dysgenesis of the eye and/or coloboma/microphthalmia (Figure 1), with syndromic features in most cases (Table 1 and Figure 2).

Individual 1 is a 5-year-old white (Canadian) male with a clinical diagnosis of a Peters plus-like syndrome. He has unilateral ocular anomalies consisting of type-2 Peters anomaly, nystagmus and a central retinal scar. Craniofacial features include discolored central incisors, microcephaly, deep set eyes, up-slanting palpebral fissures, smooth philtrum, thin upper lip, cupped ear and white locks of hair in the posterior hairline. He also has a congenital heart defect (perimembranous VSD, pulmonary stenosis), chronic lung disease, Grade 3 vesicoureteral reflux, a feeding disorder requiring g-tube, C2-C3 subluxation and right hip dislocation. Growth and development are affected with severe cognitive impairment and borderline short stature (third centile). Exome sequencing identified a likely pathogenic variant in *KMT2D*, c.1300del p.(Leu434*), which was not present in the mother (father unavailable); this variant has been reported as pathogenic in ClinVar (280135). 

Individual 2 (Figure 1A–D) is a 21-month-old white (Turkish) male with left microphthalmia, peripheral sclerocornea and esotropia and right eye abnormally shaped optic nerve (large with abnormal vessels) and retinal thinning seen in the fundoscopic exam (Figure 1D). He was able to fix and follow with the right eye only with −1.0 diopter of myopia seen via retinoscopy. Craniofacial features include microcephaly, smooth philtrum, thin upper lip, low-set cupped ears and large irregular teeth. Other systemic anomalies include a congenital heart defect (aortic stenosis), ureteral stenosis and horseshoe shaped kidney, as well as hip dislocation, hearing loss and history of feeding disorder. Growth and development are severely affected with height of 68 cm and weight of 7.7 kg (both 50th centile for a 6-month-old) and significant developmental delay (unable to sit unsupported or crawl). Trio exome sequencing identified a de novo pathogenic variant in *KMT2D*, c.14006C>G p.(Ser4669*). A second variant, c.5782+1G>A, was present in the proband, unaffected mother and unaffected sibling. While this variant has been reported as likely pathogenic in ClinVar (1506261), the allele is present in the general population in gnomAD v3.1.2 (2/68,012 European), Iranome (1/1600) and Turkish Variome (1/5174). Closer examination identified an alternative donor splice site 9-bp into the intron, so the predicted effect of the donor loss is a 3 amino acid insertion (p.(Gly1928_Gly1929insAsnThrGly)). Combined with co-segregation data in our family, this allele meets criteria to be considered likely benign. 

Individual 3 is a 14-year-old white (American) male with a clinical diagnosis of Axenfeld-Rieger syndrome. He has unilateral iris hypoplasia along with mandibular prognathism, low-set cupped ears, thin upper lip with smooth philtrum, absent central maxillary incisors, peg-shaped teeth, vertebral anomalies and mild–moderate cognitive impairment. Trio exome sequencing identified a de novo pathogenic variant in *KMT2D*, c.16294C>T p.(Arg5432Trp), which has been reported in ClinVar (633524) and three literature reports [14,15,16]. This missense change occurs within the SET domain and has very high CADD and REVEL scores.

In addition to the pathogenic/likely pathogenic variants described above, we identified two variants of uncertain significance in additional unrelated individuals with unilateral ocular coloboma and microphthalmia (Individuals 4 and 5). Both individuals had novel missense variants in disordered regions of *KMT2D*, c.6692C>T p.(Pro2231Leu) and c.14099_14105delATTCTCCinsGTTCTCT p.(Asp4700_Pro4702delinsGlySerLeu), resulting in two amino acid changes (AspSerPro to GlySerLeu) affecting the same allele. Each VUS has moderate to high CADD and REVEL scores but parental samples were not available for co-segregation studies.

Individual 6 is a 9-year-old white (American) female with Trisomy 21. Ocular anomalies include bilateral congenital cataract, nystagmus and right possible staphyloma identified via MRI. Craniofacial features include submucous cleft palate, velopharyngeal insufficiency, enamel/dentin anomalies, round face with prognathism, telecanthus, epicanthus, up-slanted palpebral fissures, horizontal eyebrows, ear anomalies (right under-folded helix, left crimped helix, bilateral small lobes), depressed nasal bridge and short columella, smooth philtrum, widely spaced teeth and downturned corners of the mouth. Other systemic anomalies include congenital heart defects (atrial septal defect and patent ductus arteriosus), feeding disorder requiring g-tube, hypothyroidism and hearing loss. Growth and development were impaired with severe hypotonia, profound delays (non-verbal; limited mobility and first walked at 7 years) and failure to thrive (<first centile on Down syndrome growth chart). Trio exome sequencing was undertaken due to the severity of clinical features. A pathogenic de novo variant in *KMT2C*, c.103del p.(Arg35Aspfs*70), was identified.

Individual 7 (Figure 1E–H) is a 2-year-old white male with a clinical diagnosis of Axenfeld-Rieger syndrome with posterior embryotoxon, mild iridocorneal adhesions on gonioscopy and pupillary defects (slit pupils as infant which resolved with daily dilation). Craniofacial features include depressed nasal bridge, cupped ears, mild micrognathia and high forehead. Other systemic anomalies included Wolff-Parkinson-White syndrome, constipation and feeding disorder which resolved with therapy. Development is delayed with hypotonia. Trio exome sequencing identified a de novo pathogenic variant in *SETD1A (KMT2F),* c.526C>T p.(Arg176*), which was also not present in the three unaffected siblings.

### 3.2. Genetic Variants Identified in Histone Lysine Demethylase Genes, KDM6A and KDM5C

Two variants in histone lysine demethylase genes were identified in unrelated families affected with syndromic anterior segment dysgenesis of the eye and/or coloboma/microphthalmia (Table 1 and Figure 2).

Individual 8 is a white female of unknown age. She has bilateral coloboma, nystagmus and left microphthalmia. Non-ocular anomalies include short stature, seizures and developmental delay. Singleton exome sequencing identified a likely pathogenic variant in *KDM6A*, c.4087C>T p.(Arg1363*). This variant was reported once in the literature with a limited phenotypic description of multiple congenital anomalies [17].

Individual 9 is a 4-month-old Hispanic (Mexican) female with bilateral Peters anomaly with peripheral anterior synechiae, partially absent Descemet’s membrane seen via histology and right glaucoma. Craniofacial features include low-set posteriorly rotated ears and high arched palate. Other systemic anomalies include a congenital heart defect (ventricular septal defect, patent ductus arteriosus and patent foramen ovale), dilation of lateral ventricles, mild dilation of the third ventricle, mild fullness of right renal system and pulmonary hypertension. Growth and development were impaired with hypotonia, developmental delay, history of intrauterine growth restriction and short stature. Trio exome sequencing identified a likely pathogenic de novo variant in *KDM5C*, c.1204G>A p.(Asp402Asn). This missense variant has a high CADD and moderate REVEL score; it occurs between known protein domains but was previously reported as likely pathogenic in ClinVar (1710153) and a similar c.1204G>T p.(Asp402Tyr) was reported with functional characterization of deleterious effects [6,18]. 

## 4. Discussion

All of the individuals reported here had features consistent with the identified congenital regulopathy, but none had the diagnosis identified clinically, likely due to the non-specific nature of many features of these conditions and/or the presence of ocular features not previously associated with these phenotypes. Interestingly, two other genetic diagnoses were commonly identified: a clinical diagnosis of Axenfeld-Rieger syndrome was made in two individuals (Individuals 3 and 7) and two had a Peters plus-like condition (Individuals 1 and 9); Individual 2 also showed phenotypic overlap with Axenfeld-Rieger syndrome. A review of the features does show substantial overlap, especially for Individual 3, with iris hypoplasia, hypertelorism and missing teeth [19]. Similarly, Peters anomaly and short stature are considered cardinal features of Peters plus syndrome, though the craniofacial features differ [20]. Overall, it seems likely that the presence of rare ocular features distracted from the phenotypic fit with a congenital regulopathy. 

While anterior segment dysgenesis disorders are not a recognized feature of congenital regulopathies, the association is not inconsistent with what is known about the genes. All of the genes reported here are expressed in the developing eye in the mouse and, in many cases, ocular expression is higher than brain expression [21]. Visual impairment is common in regulopathies, but it is usually due to strabismus or refractive errors [5,18,22]. A review of ophthalmological features of Kabuki syndrome identified ptosis, strabismus and refractive errors as common features but also reported microphthalmia, coloboma and corneal opacity occasionally [22]. Another literature review of 1369 individuals with Kabuki syndrome identified microphthalmia/ coloboma in 3% overall [3] and individuals with variants in a specific region of *KMT2D* were recently found to show overlap with features of CHARGE syndrome, including microphthalmia/coloboma in 22% of those with missense variants in exon 38/39 of *KMT2D* [23]. While developmental eye anomalies are not recognized as features of the other congenital regulopathies, a much smaller number of cases has been reported for these genes. Finally, effects on neural crest development, known to be critical for anterior segment formation, have been identified for *KMT2D* [24], *KDM6A* [25] and *KDM5C* [26], and animal models for *KDM6A* and *KDM5C* deficiency also have eye phenotypes [26,27].

The role of *KMT2C* in the congenital cataract phenotype is less clear given the coexisting diagnosis of Trisomy 21 (T21). The neurological phenotype is more severe than typically seen in either T21 or Kleefstra syndrome, likely due to the cumulative effect of T21 and the *KMT2C* variant in neurodevelopment. While congenital cataract is reported in 1–2% of children with T21 [28] and has not been reported as being part of Kleefstra syndrome, an RNA-seq study of the developing lens identified *KMT2C* as a candidate for cataract, based on enrichment in the developing lens [29]. The variant reported here is the earliest truncating variant reported to date [30], which may have a larger effect on lens development compared to prior variants. 

In summary, this study expands the phenotypic spectra associated with KMT and KDM factors and highlights the importance of genetic testing for correct clinical diagnosis, especially in the presence of rare ocular anomalies.

## Figures and Tables

**Table 1:** Summary of heterozygous genetic variants identified in histone lysine methyltransferase (KMT) and demethylase (KDM) genes.

ID	Gene	Location (hg19)	Variant Effect ^a^	CADD Score	REVEL Score	gnomAD ^b^	Read Depth	Inheritance	ACMG Criteria	Ocular	Cranio-Facial	Dental	Cardiac	GU	GI	Skeletal	DD/CI	Poor Growth	Additional Findings
Indiv 1	*KMT2D*	12:49446166-G-	c.1300del p.(L434*)	-	-	NP	14/42	NP in mother	LP (PVS1, PM2_S, PP5)	R PA, NYS and central retinal scar	+	+	+	+	+	+	+	+	Chronic lung disease
Indiv 2	*KMT2D*	12:49423253-G-C	c.14006C>G p.(S4669*)	43	-	NP	35/72	de novo	P (PVS1, PS2, PM2_S)	L MI, SC; R ON anomaly and retinal thinning	+	+	+	+	+	+	+	+	Hearing loss
	*KMT2D*	12:49436523-C-T	c.5782+1G>A p.(G1928_G1929insNTG)	-	-	2/68,012 Eu	33/59	Inherited	LB (BS4, BP5)										
Indiv 3	*KMT2D*	12:49416417-G-A	c.16294C>T p.(T5432W)	33	0.907	NP	37/76	de novo	P (PS1, PS2, PM2_S, PP3)	Iris hypoplasia	+	+	-	-	-	+	+	U	-
Indiv 4	*KMT2D*	12:49434861-G-A	c.6692C>T p.(P2231L)	26.3	0.456	NP	21/34	U	VUS (PM2_S)	L COL, MI	U	U	-	-	-	-	U	-	-
Indiv 5	*KMT2D*	12:49422990-GGAGAAT-AGAGAAC	c.14099_14105delATTCTCCinsGTTCTCT p.(N4700_P4702delinsGSL)	29.3/32	0.71/0.565	NP	34/48	U	VUS (PM2_S, PP3)	R mild MI, COL	U	U	+	-	-	-	U	-	-
Indiv 6	*KMT2C*	7:152132769-T-	c.103del p.(R35Nfs*70)	-	-	NP	23/54	de novo	P (PVS1, PS2, PM2_S)	CC, NYS, possible staphyloma	+	-	+	-	+	-	+	+	Trisomy 21, hearing loss, hypothyroidism
Indiv 7	*SETD1A (KMT2F)*	16:30974762-C-T	c.526C>T p.(R176*)	37	-	NP	29/65	de novo	P (PVS1, PS2, PM2_S)	PE, ICA, corectopia (resolved)	+	-	+	-	+	-	+	-	
Indiv 8	*KDM6A*	X:44969405-C-T	c.4087C>T p.(R1363*)	36	-	NP	75/179	U	LP (PVS1, PM2_S)	B COL and NYS, L MI	U	-	-	-	-	-	+	+	Seizures
Indiv 9	*KDM5C*	X:53241007-C-T	c.1204G>A p.(D402N)	29.7	0.481	NP	35/64	de novo	LP (PS2, PM2_S, PM5, PP2, PP5)	B PA, R GL	+	-	+	-	-	-	+	+	Pulmonary hypertension, dilated ventricles

B, bilateral; CC, congenital cataracts; COL, coloboma; GL, glaucoma; ICA, iridocorneal adhesions; L, left; MI, microphthalmia; NYS, nystagmus; ON, optic nerve; PA, Peters anomaly; PE, posterior embryotoxon; R, right; SC, sclerocornea. m, month; y, year; CI, cognitive impairment; DD, developmental delay; GI, gastrointestinal anomaly; GU: genitourinary anomaly; LP: likely pathogenic; NP: not present; P: pathogenic; U: unknown; VUS: variant of unknown significance. ^a^ KMT2D: NM_003482.4; KMT2C: NM_170606.2; SETD1A: NM_014712.3; KDM6A: NM_021140.4; KDM5C: NM_004187.5. ^b^ gnomAD v3.1.2.

## Data Availability

All variants were submitted to ClinVar. There are no additional data available.
